# The Inhibitory Activities and Antiviral Mechanism of Medicinal Plant Ingredient Quercetin Against Grouper Iridovirus Infection

**DOI:** 10.3389/fmicb.2020.586331

**Published:** 2020-10-16

**Authors:** Mingzhu Liu, Qing Yu, Hehe Xiao, Mengmeng Li, Yaming Huang, Qin Zhang, Pengfei Li

**Affiliations:** ^1^ Guangxi Key Laboratory of Marine Natural Products and Combinatorial Biosynthesis Chemistry, Guangxi Beibu Gulf Marine Research Center, Beibu Gulf Marine Industrial Research Institute, Guangxi Academy of Sciences, Nanning, China; ^2^ College of Life Science, Henan Normal University, Xinxiang, China; ^3^ Guangxi Key Laboratory for Polysaccharide Materials and Modifications, Guangxi Colleges and Universities Key Laboratory of Utilization of Microbial and Botanical Resources, School of Marine Science and Biotechnology, Guangxi University for Nationalities, Nanning, China; ^4^ Guangxi Key Laboratory of Beibu Gulf Marine Biodiversity Conservation, College of Marine Sciences, Beibu Gulf University, Qinzhou, China

**Keywords:** natural ingredient quercetin, medicinal plant, *Illicium verum* Hook. f., iridovirus, antiviral effects and mechanism, aquaculture

## Abstract

Singapore grouper iridovirus (SGIV) causes high mortality rates in mariculture, and effective treatments against SGIV infection are urgently required. *Illicium verum* Hook. f. (*I. verum*) is a well-known medicinal plant with a variety of biological activities. The natural ingredient quercetin isolated from *I. verum* could effectively inhibit SGIV infection in a dose-dependent manner. The possible antiviral mechanism of quercetin was further analyzed in this study. It showed that quercetin did obvious damages to SGIV particles. Furthermore, quercetin could interfere with SGIV binding to targets on host cells (by 76.14%), disturb SGIV invading into host cells (by 56.03%), and effect SGIV replication in host cells (by 52.73%), respectively. Quercetin had the best antiviral effects during the SGIV life cycle of binding to the receptors on host cells’ membranes. Overall, the results suggest that quercetin has direct and host-mediated antiviral effects against SGIV and holds great potential for developing effective drugs to control SGIV infection in aquaculture.

## Introduction

Grouper (*Epinephelus* spp.) is an economically important mariculture species, and the aquaculture production has reached 159,579 tons in 2018 (Zhang et al., 2019). However, with the rapidly expanding scale and density of grouper aquaculture, the aquatic environment is deteriorating day by day, which results in the increasing incidence of serious pathogen outbreaks and threatens the sustainable development of grouper aquaculture (Li et al., 2018; Yu et al., 2019a). *Iridovirus* is one of the most serious pathogenic viruses in cultured fish and has been isolated from more than 100 fish species worldwide. The Singapore grouper iridovirus (SGIV) belonged to *Iridovirus* and was isolated from diseased groupers (Qin et al., 2003; Xiao et al., 2019). Some typical clinical signs of SGIV infection, such as spleen and liver enlargement and large-scale mortality, occur within 1 or 2 weeks. Therefore, effective anti-SGIV drugs are urgently needed.

Medicinal plants have been applied to treat numerous diseases for thousands of years and are being increasingly used in aquaculture (Khan et al., 2005; Gezici and Şekeroğlu, 2019). Medicinal plants are known as “green medicine,” with some advantages over chemically synthesized medicines: (1) medicinal plants provide eco-friendly compounds for replacing antibiotics, (2) medicinal plants are used in different forms, including aqueous extracts, ethanol extracts, and active ingredients, which could serve as immunostimulants to prevent and control pathogens; furthermore, medicinal plants at different concentrations and through different ways, such as injection or immersion or oral administration, could cause various levels of immune stimulation, (3) medicinal plants have positive effects on cultured fish, including increasing digestive enzyme activities, stimulation of growth, weight gain, early maturation, and meat quality, and (4) medicinal plants could obviously inhibit pathogens (bacteria, virus, and parasite) and medicinal plants with immunostimulatory and antiviral properties applied in aquaculture have attracted increasing attention (Mukhtar et al., 2008; Bulfon et al., 2015; Hai, 2015; Newaj-Fyzul and Austin, 2015; Awad and Awaad, 2017; Yu et al., 2019b). For example, licorice is one common medicinal plant that has been used for centuries. There have been about 300 flavonoids and more than 20 triterpenoids isolated from licorice, which possess many pharmacological activities, such as antimicrobial, antiviral, and antitumor activities (Wang et al., 2015). Ma et al. (2002) generated 27 of 44 medicinal plants, which showed potent or moderate antiviral activities against the respiratory syncytial virus (RSV), and further identified some active extracts (anagyrine, oxymatrine, sophoranol, wogonin, and oroxylin A) as potent anti-RSV components (Ma et al., 2002). Moreover, the total flavonoids extracted from *Lonicera japonica* Thunb have effective anti-influenza activity against H9N2 (Wang et al., 2006).

*Illicium verum* Hook. f. (*I. verum*) is a famous medicinal plant since the ancient times and known as “drug homologous food” in China. *I. verum* possesses abundant active ingredients, including flavonoids, organic acids, and terpenoids, which endow *I. verum* with numerous biological activities, including anti-inflammatory, antimicrobial, antiviral, and anticancer effects; these activities are usually associated with low toxicity, making *I. verum* a promising medicinal plant source of therapeutic drugs (Ebani et al., 2018). For example, essential oils isolated from *I. verum* could fight against herpes simplex virus (HSV) type 2 with inhibitory concentrations (IC50) at 0.016% (Koch et al., 2008). *I. verum* sesquiterpenes showed antiviral effects against hepatitis B virus (Liu et al., 2015) and HIV (Song et al., 2007). In a previous study, some *I. verum* extracts were isolated and used to treat SGIV infection *in vitro* and *in vivo*. The *I. verum* active ingredients quercetin, shikimic acid, trans-anethole, and 3,4-dihydroxybenzoic acid have dose-dependent antiviral activities against SGIV infection. Furthermore, quercetin (50 μg/ml) has the greatest antiviral activity, with percent inhibition of 99.83%, making it a potential candidate for developing effective drugs for controlling SGIV infection (Liu et al., 2020a). In this study, to address the urgent need for therapeutic agents against SGIV in aquaculture, the effects of *I. verum* ingredient quercetin against SGIV infection were assessed *in vitro*. The possible antiviral mechanisms of quercetin were analyzed.

## Materials and Methods

### Cells, Virus, and Reagents

Grouper spleen (GS) cells were maintained in Leibovitz’s L15 medium (Gibco, Grand Island, NY, USA) at 28°C (Qin et al., 2006). SGIV was isolated from the hybrid grouper (*Epinephelus fuscoguttatus*♀ × *Epinephelus lanceolatus*♂) and used in this study (Xiao et al., 2019). SGIV was purified by sucrose gradient ultracentrifugation, as described previously (Li et al., 2014). Primary antibodies against major capsid protein (MCP) were prepared from mice immunized with MCP. Quercetin was isolated from *I. verum* (>98% purity). Quercetin was diluted to a safe working concentration (50 μg/ml) with L15 medium (pH 7.5), as reported previously (Liu et al., 2020a).

### Cytotoxic Analysis of Quercetin at a Working Concentration

GS cells were seeded in a 96-well plate at 28°C for 24 h and then incubated with quercetin (50 μg/ml) in 100 μl L15 medium at 28°C for 48 h. Normal GS cells without quercetin incubation served as the control group. Then, 10 μl of CCK-8 solution (Beyotime, Shanghai, China) was added to the cells at 28°C for 4 h. Absorbance at 450 nm was measured by the ELISA plate reader (Thermo, Waltham, MA, USA). Results are presented as mean ± SD of three independent experiments.

### Fluorescence Observation for Cytoskeleton

The effects of quercetin, at a safe working concentration, on the cell were identified by fluorescence observation (Ndozangue-Touriguine et al., 2008; Su et al., 2019). GS cells (1 × 10^4^) were cultured in 35-mm glass-bottom dishes (Cellvis, catalog number D35-14-1-N) at 28°C for 24 h and then incubated with quercetin (50 μg/ml) for 24 h at 28°C. After incubation, the cells were washed with phosphate-buffered saline (PBS; pH 7.4) three times and subsequently dyed with fluorescein isothiocyanate (FITC)-labeled anti-vimentin antibody, FITC-labeled anti-cytokeratin antibody, and FITC-labeled anti-fibronectin antibody (Abcam, UK), respectively. Fluorescence was observed by laser scanning confocal microscopy (LSCM, Nikon, C2, Japan). Normal GS cells without quercetin treatment served as the control group.

### Virus Fluorescence Labeling

Virus fluorescence labeling was performed as previously described (Yu et al., 2019c). Purified SGIV particles were incubated with dye Cy5 at room temperature for 2 h with gentle vortexing. The unbound dye was removed and washed by three high-speed centrifugations at 42,000 *g* at 4°C for 1 h. Cy5-labeled SGIV (Cy5-SGIV) pellets were suspended in PBS and passed through 0.22-μm-pore-size filters and examined by transmission electron microscopy.

### Confirmation of Gene Expression *via* RT-qPCR

The cells and the culture medium in each well were collected for total RNA extraction. Total RNA was then reverse-transcribed into cDNA with ReverTra Ace® qPCR RT Kit (Toyobo, Osaka, Japan). SGIV infection was identified by detecting SGIV MCP and viral envelope protein (VP19) transcripts with RT-qPCR, as previously described (Liu et al., 2019, 2020b). The β-actin gene was used as an internal control. The primers used for qRT-PCR are listed in Table 1. The results from three independent experiments were presented as mean ± SD.

**Table 1:** The primers used for detecting Singapore grouper iridovirus (SGIV) infection in qRT-PCR.

Primer	Sequences
qMCP-F	5'-GCACGCTTCTCTCACCTTCA-3'
qMCP-R	5'-AACGGCAACGGGAGCACTA-3'
qVP19-F	5'-TCCAAGGGAGAAACTGTAAG-3'
qVP19-R	5'-GGGGTAAGCGTGAAGACT-3'
β-actin-F	5'-TACGAGCTGCCTGACGGACA-3'
β-actin-R	5'-GGCTGTGATCTCCTTCTGCA-3'

### Western Blot Assay

GS cells were treated with quercetin (50 or 25 μg/ml) and SGIV at 28°C for 48 h. At 48 h post-infection (hpi), the cells and the culture supernatants were collected for protein extraction. Proteins were separated in sodium dodecyl sulfate-polyacrylamide gel electrophoresis (12%) and then transferred to polyvinylidene fluoride membrane. The blots were blocked for 2 h at room temperature with 5% skim milk in Tris-buffered saline (TBS; 0.1% Tween 20) and incubated with anti-MCP antibodies (1:500). The blots were washed with TBS and incubated with secondary antibodies (1:1,000). The blots were reprobed with α-tubulin (1:1,000) for total protein.

### Characterization of SGIV Infection by Virus Titer

The cells and the culture medium were collected and transferred to a 96-well plate to determine virus titer by 50% tissue culture infection dose (TCID_50_) assay, as previously described (Li et al., 2014). Briefly, GS cells were cultured in a 12-well plate (Corning) for 24 h at 28°C, after which quercetin (50 or 25 μg/ml) and SGIV, at a multiplicity of infection (MOI) of 0.1, were simultaneously added to the cells. GS cells added with only SGIV (MOI = 0.1) served as the control group. The cells were maintained at 28°C for 48 h. At 48 hpi, the cells and the culture supernatants were collected for virus titer analysis. The data from three independent experiments were used to quantify the effects of quercetin on SGIV infection.

### Antiviral Mechanism of Quercetin Against SGIV Infection: Effects of Quercetin on SGIV Particles

The antiviral mechanism of quercetin against SGIV infection was further analyzed, as previously described with modification (Liu et al., 2017; Yu et al., 2019b). Then, 100 μl SGIV (10^7^ TCID_50_/ml) was mixed with quercetin (50 μg/ml) at 4°C for 2 h. After having been centrifuged at 42,000 *g* at 4°C for 1 h, virion pellets were collected and suspended in 100 μl TN buffer. Furthermore, 10 μl SGIV was added into GS cells in a 12-well plate at 28°C. At 48 hpi, the cells and the culture medium in each well were collected for total RNA extraction and analyzed by RT-qPCR. SGIV without incubation with quercetin (50 μg/ml) served as the control group, and SGIV in the control group was processed as per the same procedures.

### Antiviral Mechanism of Quercetin Against SGIV Infection: Effects of Quercetin on SGIV Binding to Host Cells

The effects of quercetin on SGIV binding to host cells were first analyzed by RT-qPCR. GS cells were seeded in a 12-well plate at 28°C for 24 h. Quercetin (50 μg/ml) was incubated with GS cells at 4°C for 30 min. The culture supernatants were removed, and the cells were washed twice with L15. Cells were incubated with SGIV (MOI = 0.1) at 4°C for 30 min and then washed twice with L15. The cells and the culture medium in each well at 12 hpi were collected for total RNA extraction and SGIV infection detection by RT-qPCR. GS cells infected with SGIV only (MOI = 0.1) served as the control group.

The effects of quercetin on SGIV binding to host cells were also analyzed by flow cytometry analysis. GS cells in a 12-well plate were incubated with quercetin (50 μg/ml) at 4°C for 30 min. Then, Cy5-labeled SGIV (Cy5-SGIV, MOI = 1) was added to the cells at 4°C for 30 min. The cells were washed twice with PBS and collected for flow cytometry analysis. GS cells infected with Cy5-SGIV only (MOI = 1) served as the control group.

The effects of quercetin on SGIV binding to host cells were further analyzed by LSCM. For live-cell fluorescent imaging, GS cells in 35-mm glass-bottom dishes (Cellvis, Hangzhou, Zhejiang, China) were incubated with quercetin (50 μg/ml) at 4°C for 30 min. Then, Cy5-labeled SGIV (Cy5-SGIV, MOI = 1) was added to the cells at 4°C for 30 min. After having been washed with serum-free, phenol-red-free medium, their fluorescence was detected by LSCM. GS cells infected with Cy5-SGIV only (MOI = 1) served as the control group.

### Antiviral Mechanism of Quercetin Against SGIV Infection: Effects of Quercetin on SGIV Invading Into Host Cells

The effects of quercetin on SGIV invading into host cells were first analyzed by RT-qPCR. GS cells were seeded in a 12-well plate at 28°C for 24 h. Cells were incubated with SGIV (MOI = 0.1) at 4°C for 30 min, which made SGIV bind to the cell surface. After having been washed twice with L15, quercetin (50 μg/ml) was added to the cells and incubated at 28°C for 2 h. The culture supernatants were removed, and cells were washed twice with L15. GS cells were cultured at 28°C. The cells and the culture medium in each well at 12 hpi were collected for total RNA extraction and SGIV infection detection by RT-qPCR. Cells without quercetin treatment served as the control group.

The effects of quercetin on SGIV invading into the host cells were also analyzed by flow cytometry analysis. GS cells in a 12-well plate were infected with Cy5-SGIV (MOI = 1) at 4°C for 30 min. After having been washed twice with PBS, the cells were incubated with quercetin (50 μg/ml) at 28°C for 2 h. GS cells incubated with Cy5-SGIV only (MOI = 1) served as the control group. To remove the cellular surface’s virus particles, the cells in each group were then digested in protease K for 2 min at 28°C, respectively. After having been washed three times with PBS, the cells were collected for flow cytometry analysis.

The effects of quercetin on SGIV invading into the host cells were further analyzed by LSCM. For live-cell fluorescent imaging, GS cells in 35-mm glass-bottom dishes were infected with Cy5-SGIV (MOI = 1) at 4°C for 30 min. After having been washed twice with PBS, the cells were incubated with quercetin (50 μg/ml) at 28°C for 2 h. GS cells incubated with Cy5-SGIV only (MOI = 1) served as the control group. After having been washed with serum-free, phenol-red-free medium, their fluorescence was further detected by LSCM.

### Antiviral Mechanism of Quercetin Against SGIV Infection: Effects of Quercetin on SGIV Replication in Host Cells

GS cells were seeded in a 12-well plate at 28°C for 24 h. Cells were incubated with SGIV (MOI = 0.1) at 4°C for 30 min and then maintained at 28°C for 2 h to make SGIV enter the host cells. The culture supernatants were removed, and the cells were washed twice with L15. Quercetin (50 μg/ml) was added and incubated with GS cells at 28°C for 10 h. SGIV-infected cells without quercetin treatment served as the control group. The cells and the culture medium in each well at 12 hpi were collected for SGIV replication analysis by RT-qPCR and virus titer detection. The cells and the culture medium in each well at 24 hpi were collected for SGIV replication analysis by virus titer detection.

### Inhibitory Percentage of Quercetin on a Different Stage of SGIV Infection

The inhibitory percentage of quercetin on a different stage of SGIV infection was evaluated as described previously (Yu et al., 2019b). Percentage inhibition = 1 − (X − B)/(A − B) × 100%. X represents each RT-qPCR results of cells treated with SGIV and quercetin on a different stage of SGIV infection, A represents the RT-qPCR result for cells treated with SGIV alone, and B represents the RT-qPCR result of normal cells.

### Statistical Analysis

The average value of three independent experiments was calculated. Intergroup differences were compared using one-way analysis of variance with SPSS statistical software (IBM, Armonk, NY, USA). The results of comparisons with *p* < 0.05 were considered to represent statistically significant differences.

## Results

### Quercetin at a Working Concentration Exhibited No Cytotoxic Effects

**Figure 1:**
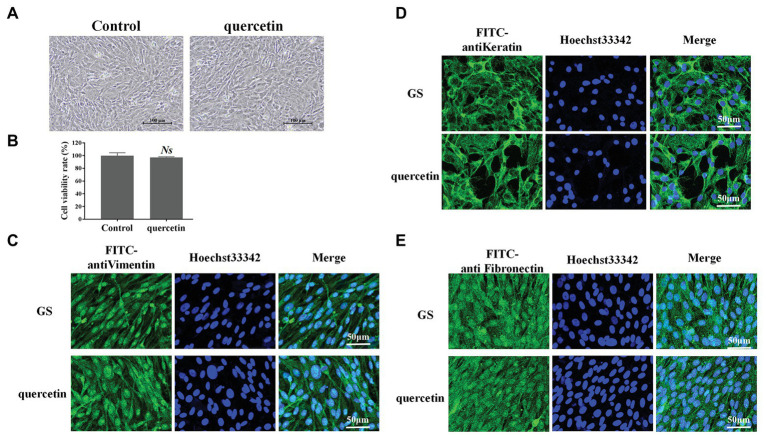
Quercetin at working concentration exhibited no cytotoxic effects. **(A)** Grouper spleen (GS) cells were incubated with quercetin (50 μg/ml) for 24 h and observed under a light microscope. GS cells incubated with quercetin kept a normal growth. Scale bar: 100 μm. **(B)** The cell viability of GS cells was incubated with quercetin (50 μg/ml) using the CCK-8 solution. Quercetin (50 μg/ml) displayed no significant cytotoxic effects. *NS* indicates no statistical significance. **(C–E)** Fluorescein isothiocyanate-labeled anti-vimentin, anti-cytokeratin, and anti-fibronectin antibodies were applied to observe the cytoskeleton. Compared with cells in the control group, the cytoskeleton of cells incubated with quercetin (50 μg/ml) was kept normal. Scale bar: 50 μm.

The possible cytotoxic effects of quercetin on GS cells were evaluated after 24 h of incubation. GS cells were first observed under a light microscope (Figure 1A). Compared with normal cells in the control group, GS cells incubated with quercetin (50 μg/ml) kept normal growth; there were no pathological and morphological changes (Figure 1A). The cell viability of GS cells incubated with quercetin (50 μg/ml) was further evaluated using CCK-8 solution, and it was consistent with light microscopy observation results (Figure 1B). The cytoskeleton is a major mechanical structure of a cell and plays an important role in cell functions (Fletcher and Mullins, 2010). FITC-labeled anti-vimentin, anti-cytokeratin, and anti-fibronectin antibodies were applied to observe the cytoskeleton (Figures 1C–E). It showed that, compared with cells in the control group, the cytoskeleton of cells incubated with quercetin (50 μg/ml) was kept normal, suggesting that safe concentrations of quercetin did not cause any cytotoxic effects. These results were consistent with observations in a previous study which showed that quercetin, at a safe working concentration (≤50 μg/ml), has no cytotoxicity on cultured GS cells (Liu et al., 2020a).

### Inhibition Effects of Quercetin on SGIV Infection

**Figure 2:**
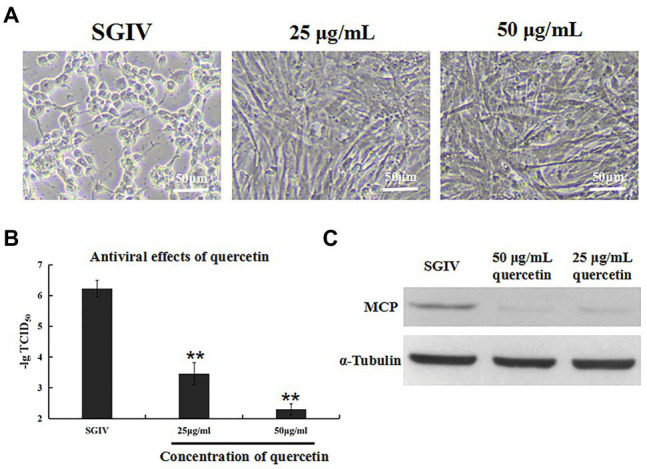
Inhibition effects of quercetin on SGIV infection. GS cells incubated with SGIV and quercetin (50 or 25 μg/ml) served as the test group; cells infected with SGIV alone served as the control group. **(A)** Cell morphology was observed by light microscopy. Compared to large numbers of typical cytopathic effects (CPEs) appearing in the control group, few CPEs appeared in cells incubated with SGIV and quercetin (50 or 25 μg/ml). **(B)** Compared to the control group, quercetin could significantly reduce virus titers at 48 hpi in a dose-dependent manner. **(C)** The protein levels of major capsid protein (MCP) in SGIV-infected GS cells increased significantly after quercetin treatments. ^**^ *p* < 0.01.

The antiviral activity of quercetin against SGIV infection was analyzed by cell morphology observation and virus titer (Figure 2). GS cells incubated with SGIV and quercetin (50 or 25 μg/ml) were the test group, and cells infected with SGIV alone served as the control group. As shown in Figure 2A, cell morphology was observed by light microscopy. Compared to large numbers of typical cytopathic effects (CPEs) appearing in the control group, few CPEs appeared in cells incubated with SGIV and quercetin (50 or 25 μg/ml). Furthermore, compared to the control group, quercetin could significantly reduce virus titers at 48 hpi in a dose-dependent manner (Figure 2B). Similarly, the protein levels of MCP in SGIV-infected GS cells increased significantly after quercetin treatments (Figure 2C).

### Antiviral Mechanism of Quercetin Against SGIV Infection: Damaging Effects of Quercetin on SGIV Particles

**Figure 3:**
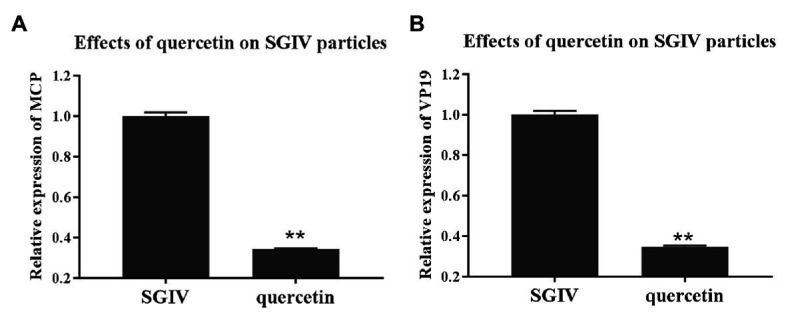
Quercetin had damaging effects on SGIV particles. GS cells incubated with quercetin-treated SGIV served as the test group, and cells infected with SGIV alone served as the control group. Compared to the control group, the levels of viral MCP **(A)** and viral envelope protein **(B)** gene expression in the test group’s cells were lower than those in the control group. It indicated that quercetin had damaging effects on SGIV particles (^**^ *p* < 0.01).

To explore quercetin’s antiviral mechanism against SGIV infection, we first evaluated the damaging effects of quercetin on SGIV particles. GS cells incubated with quercetin-treated SGIV were the test group, and cells infected with SGIV alone served as the control group. As shown in Figure 3, the levels of viral MCP and VP19 gene expressions in the cells of the test group were lower than those in the control group of cells infected with SGIV alone, indicating that quercetin had damaging effects on SGIV particles.

### Inhibitory Effects of Quercetin on SGIV Binding to Host Cells

**Figure 4:**
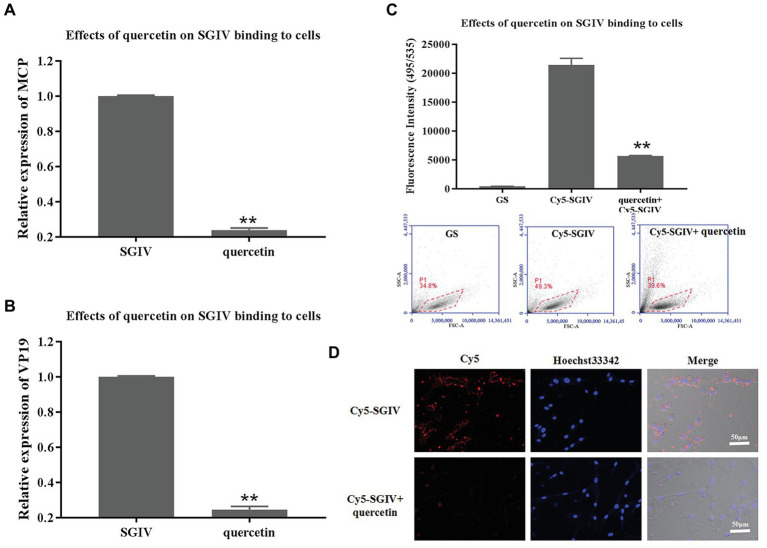
Inhibitory effects of quercetin on SGIV binding to host cells. **(A,B)** The inhibitory effects of quercetin on SGIV binding to host cells were analyzed by RT-qPCR. Test group: GS cells pretreated with quercetin at 4°C for 30 min and then infected with SGIV at 28°C for 12 h; control group: cells infected with SGIV alone for 12 h. The cells and the culture medium in each well at 12 hpi were collected for total RNA extraction and SGIV infection detection by RT-qPCR. The levels of viral MCP **(A)** and viral envelope protein **(B)** gene expression in the test group were lower than those in the control group. **(C)** The effects of quercetin on SGIV binding to host cells were analyzed by flow cytometry analysis. After having been pretreated with quercetin (50 μg/ml) at 4°C for 30 min, cells were incubated with Cy5-SGIV [multiplicity of infection (MOI) = 1] at 4°C for 30 min and then collected for flow cytometry analysis. Cells infected with Cy5-SGIV (MOI = 1) alone for 30 min served as the control group. Compared to the control group, the fluorescence signals of Cy5 on the cells of the test group decreased obviously. It proved that quercetin did interfere with SGIV binding to targets on host cells (^**^ *p* < 0.01). **(D)** The effects of quercetin on SGIV binding to host cells were further analyzed by laser scanning confocal microscopy (LSCM). The LSCM observation results were consistent with the flow cytometry results. Compared to the control group, the fluorescence signals of Cy5 on the surface of cells in the test group decreased obviously. Scale bar: 50 μm.

The effects of quercetin on SGIV binding to host cells were first analyzed by RT-qPCR. GS cells pretreated with quercetin and then infected with SGIV were the test group; cells infected with SGIV alone served as the control group. As shown in Figures 4A,B, the levels of viral MCP and VP19 gene expression in the cells of the test group were lower than those in the control group of cells infected with SGIV alone (Figures 4A,B). The inhibitory effects of quercetin on SGIV binding to the host cell surface were also proven by flow cytometry analysis (Figure 4C). After having been pretreated with quercetin (50 μg/ml) at 4°C for 30 min, the cells were incubated with Cy5-SGIV (MOI = 1) at 4°C for 30 min and then collected for flow cytometry analysis. Cells infected with Cy5-SGIV (MOI = 1) alone served as the control group. As shown in Figure 4C, compared to the control group, the fluorescence signals of Cy5 on the cells of the test group decreased obviously, which proved that quercetin did interfere with SGIV binding to the targets on host cells (Figure 4C). The effects of quercetin on SGIV binding to host cells were further analyzed by LSCM (Figure 4D). The LSCM observation results were consistent with the flow cytometry results. Compared to the control group, the fluorescence signals of Cy5 on the surface of cells in the test group decreased.

### Inhibitory Effects of Quercetin on SGIV Invading Into the Host Cells

**Figure 5:**
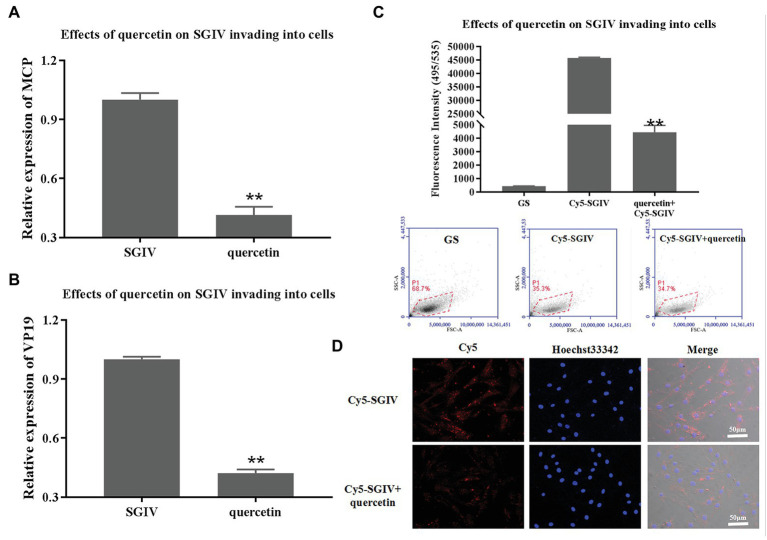
Inhibitory effects of quercetin on SGIV invading into host cells. **(A,B)** The inhibitory effects of quercetin on SGIV invading into host cells were analyzed by RT-qPCR. Test group: SGIV-infected cells at 0–2 hpi treated with quercetin; control group: SGIV-infected cells without quercetin treatment. The cells and the culture medium in each well at 12 hpi were collected for RT-qPCR analysis. The levels of viral MCP **(A)** and viral envelope protein **(B)** gene expression in the test group were lower than those in the control group. **(C)** The inhibitory effects of quercetin on SGIV invading into host cells were proven by flow cytometry analysis. After having been incubated with Cy5-SGIV [multiplicity of infection (MOI) = 1] at 4°C for 30 min, the cells were treated with quercetin (50 μg/ml) at 28°C for 2 h. Cells infected with Cy5-SGIV (MOI = 1) alone served as the control group. The cells in each group were then digested in protease K and collected for flow cytometry analysis. Compared to the control group, the fluorescence signals of Cy5 on cells of the test group decreased obviously. It proved that quercetin had inhibitory effects on SGIV invading into host cells (^**^ *p* < 0.01). **(D)** Quercetin’s effects on SGIV invading into host cells were further identified by LSCM. The LSCM observation results were consistent with the flow cytometry results, which showed that the fluorescence signals of Cy5 in the cells of the test group decreased obviously. Scale bar: 50 μm.

SGIV-infected cells at 0–2 hpi treated with quercetin were the test group; SGIV-infected cells without quercetin treatment served as the control group. The cells and the culture medium in each well at 12 hpi were collected for RT-qPCR analysis. As shown in Figures 5A,B, the levels of viral MCP and VP19 gene expression in the cells of the test group decreased obviously (Figures 5A,B). The inhibitory effects of quercetin on SGIV invading into the host cells were also proven by flow cytometry analysis (Figure 5C). After having been incubated with Cy5-SGIV (MOI = 1) at 4°C for 30 min and washed with PBS, the cells were treated with quercetin (50 μg/ml) at 28°C for 2 h. Cells infected with Cy5-SGIV (MOI = 1) alone served as the control group. The cells in each group were then digested in Protease K and collected for flow cytometry analysis. As shown in Figure 5C, compared to the control group, the fluorescence signals of Cy5 on the cells of the test group decreased obviously, which proved that quercetin had inhibitory effects on SGIV invading into the host cells (Figure 5C). The effects of quercetin on SGIV invading into the host cells were further identified by LSCM (Figure 5D). The LSCM observation results were consistent with the flow cytometry results, which showed that the fluorescence signals of Cy5 in the cells of the test group decreased obviously.

### Inhibitory Effects of Quercetin on SGIV Replication in Host Cells

**Figure 6:**
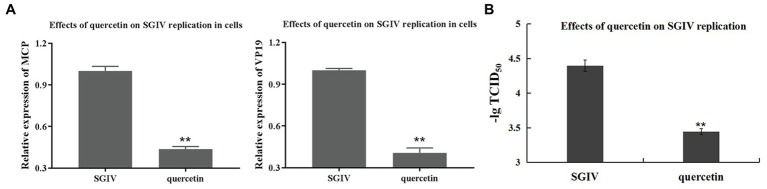
Inhibitory effects of quercetin on SGIV replication in host cells. **(A)** Inhibitory effects of quercetin on SGIV binding to host cells were analyzed by RT-qPCR. Test group: SGIV-infected cells at 2 hpi treated with quercetin at 28°C for 10 h; control group: SGIV-infected cells without quercetin treatments. **(A)** The levels of viral MCP and VP19 gene expression in the cells of test groups decreased obviously. **(B)** Inhibitory effects of quercetin on SGIV replication in host cells were proved by virus titer analysis. Compared to the control group, quercetin could significantly reduce virus titers. It suggested that SGIV replication in host cells could be inhibited by quercetin (^**^ *p* < 0.01).

The effects of quercetin on SGIV replication in the host cells were analyzed by RT-qPCR and virus titer (Figure 6). SGIV-infected cells at 2 hpi and treated with quercetin for 10 h were the test group; SGIV-infected cells without quercetin treatment served as the control group. As shown in Figure 6A, the levels of viral MCP and VP19 gene expression in the cells of the test group decreased obviously (Figure 6A). The inhibitory effects of quercetin on SGIV replication in the host cells were also proven by virus titer analysis (Figure 6B). Compared to the control group, quercetin could significantly reduce virus titers at 24 hpi, which suggested that SGIV replication in the host cells could be inhibited by quercetin (Figure 6).

### Inhibitory Percentage of Quercetin on Different Stages of SGIV Infection

**Figure 7:**
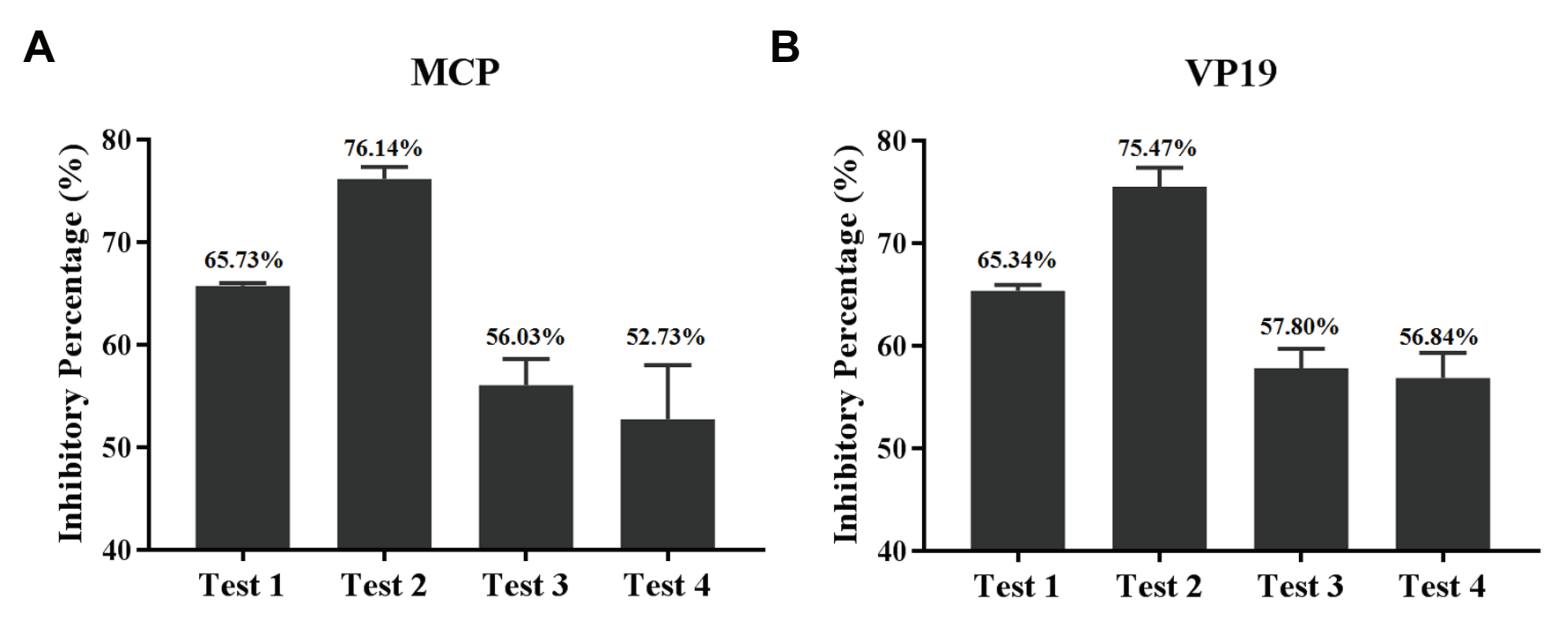
Inhibitory percentage of quercetin on a different stage of SGIV infection. **(A)** The inhibitory percentages are based on MCP gene expression. The inhibitory percentages of quercetin on SGIV particles (test 1), SGIV binding to the membrane of host cells (test 2), SGIV invading into host cells (test 3), and SGIV replication (test 4) were 65.73, 76.14, 56.03, and 52.73%, respectively. **(B)** The inhibitory percentages based on the viral envelope protein gene were consistent with those of the MCP gene.

The inhibitory percentage of quercetin on different stages of SGIV infection was evaluated by RT-qPCR results. The results based on MCP gene expression revealed that the inhibitory percentages of quercetin on SGIV particles (test 1), SGIV binding to the membrane of host cells (test 2), SGIV invading into host cells (test 3), and SGIV replication (test 4) were 65.73, 76.14, 56.03, and 52.73%, respectively (Figure 7A). The results based on the VP19 gene were consistent with those of the MCP gene (Figure 7B). Altogether quercetin had the best most effective antiviral activity against SGIV infection during virus binding to the receptor molecules on host cells’ membranes.

## Discussion

As an important medicinal plant, numerous compounds have been isolated and identified from *I. verum* Hook. f., including flavonoids, volatiles, seco-prezizaane-type sesquiterpenes, lignans, phenylpropanoids, and so on. Modern pharmacology studies have proved that these active compounds possess diverse pharmacological actions, including antiviral, antimicrobial, insecticidal, analgesic, antioxidant, sedative, and convulsive activities (Chaubey, 2008; Dzamic et al., 2009; Wang et al., 2011; Liu et al., 2020b). Some *I. verum* ingredients especially have excellent antiviral effects, such as sesquiterpenes against hepatitis B virus and HIV (Song et al., 2007; Liu et al., 2015), essential oils against HSV type 2 (Koch et al., 2008), flavonoids quercetin against Zika virus, SARS-associated coronavirus, hepatitis C virus (HCV), and influenza A virus (Park et al., 2012; Wu et al., 2015; Wong et al., 2017). In the previous study, we explored the antiviral effects of some *I. verum* extracts against SGIV. The results showed that the *I. verum* active ingredients quercetin, shikimic acid, trans-anethole, and 3,4-dihydroxybenzoic acid could combat SGIV infection. Furthermore, quercetin, at a working concentration of 50 μg/ml, has the best antiviral effects, whose inhibitory percentage reached 99.83% (Liu et al., 2020a). The quercetin antiviral mechanisms were investigated in this study to obtain valuable insights into the development of effective antiviral therapeutics against SGIV.

Fish cell lines serve as alternatives to experimental animals and have been widely used to evaluate the toxicity (Li et al., 2016). The cytoskeleton is an interconnected network of filamentous polymers and regulatory proteins composed of different types of actin, microtubules, and intermediate filaments. The cytoskeleton is responsible for cell shape, whole-cell motility, and cell organelle motility, thus playing an important role in various cell functions. Cytoskeleton defects, including alterations in microtubule stability, in axonal transport, and in actin dynamics, have been characterized in diseased cells (Wang et al., 2014; Eira et al., 2016; Liu et al., 2017). For example, the cytoskeleton in host cells plays a key role during virus infection. Spring viremia of carp virus (SVCV) infection could induce the collapse of the cytoskeletal fiber system, ring-shape structures, and filament depolymerization (Liu et al., 2017). Wang et al. (2014) first proved that SGIV invaded the host cells *via* the clathrin-mediated endocytic pathway.

Furthermore, SGIV particles are transported along both actin filaments and microtubules in host cells, and intracellular SGIV motility could be remarkably affected by the depolymerization of actin filaments or microtubules (Wang et al., 2014). In this study, quercetin’s possible cytotoxic effects were evaluated on GS cells by fluorescence observation of the cytoskeleton. As the cytoskeleton of cells incubated with quercetin (50 μg/ml) was kept normal, it proved that the safe working concentration of quercetin (50 μg/ml) had no cytotoxic effects, which was consistent with the results of the cell morphology and cell viability assay in the previous study (Liu et al., 2020a).

The study on the mechanisms of medicinal plant ingredients against virus infection could help develop effective and safe antiviral drugs. A virus infection starts with the viruses attaching to the host cell membrane, invading into the host cell. In the cells, viruses are transported to the replication site, where it initiates the expression of virus genes and replication (Wang et al., 2014; Yu et al., 2019c, 2020). As reported previously, most antivirus medicines are designed to disturb the different stages of the virus infection process. *I. verum* is the main industrial source of shikimic acid. Shikimic acid is the primary ingredient of oseltamivir phosphate, which serves as neuraminidase inhibitor and is the effective antiviral drug for the treatment and prevention of influenza (Hu et al., 2019). *Lonicera japonica* Thunb. (*L. japonica*) is an important medicinal plant worldwide. *L. japonica* is rich in chlorogenic acid, cryptochlorogenic acid, isochlorogenic acid, caffeic acid, etc.

Chlorogenic acid has been applied to treat viral upper respiratory tract infections caused by the influenza virus, respiratory syncytial virus, and parainfluenza virus. Ding et al. (2017) first elucidated the underlying mechanisms of chlorogenic acid against the influenza A virus. It indicated that chlorogenic acid could inhibit the influenza virus during the late stage of virus infection by down-regulating nucleoprotein expression. Furthermore, chlorogenic acid had neuraminidase activity and could block the release of the newly assembled virus from infected host cells (Ding et al., 2017). Liu et al. (2020b) evaluated the inhibitory activities of *L. japonica* components against SGIV infection and proved that some *L. japonica* components (isochlorogenic acid A, isochlorogenic acid B, isochlorogenic acid C, caffeic acid, luteolin, and inositol) exhibited an antiviral activity against SGIV infection in a dose-dependent manner. Liu et al. (2017) first proved the antiviral abilities of coumarin derivatives against SVCV infection. The other antiviral mechanism results indicated that coumarins (B4 and C2) did not affect the early stage of viral infection, such as virus adhesion and delivery from the endosomes to the cytosol, but exerted antiviral effects by preventing the cell apoptosis and microfilament organization disruption caused by SVCV infection. They also could up-regulate anti-oxidative enzyme gene expression and keep the balance of intracellular redox state during virus infection (Liu et al., 2017).

In the previous study, quercetin was isolated from *I. verum* and proved to combat SGIV infection with an inhibitory percentage above 99.83% at a safe working concentration of 50 μg/ml (Liu et al., 2020a). Quercetin meets the key requirements for developing commercially available drugs against SGIV infection in aquaculture. Systemic studies were performed to explore the anti-SGIV mechanisms of quercetin in this study. The virus life cycle is initiated with the virus attaching to the cell membrane, invasion into the host cell, virus replication in cells, and release of newly assembled virions from the host cells. Knowledge on how antiviral agents exert antiviral effects is essential to develop antiviral medicines (Wang et al., 2014; Yu et al., 2020). As the complete viral structure is essential for viral infection, we first evaluated quercetin’s effects on SGIV particles. It showed that pre-incubation with quercetin could reduce the virus’ infectivity. Then, we could reasonably conclude that quercetin did have damaging effects on SGIV particles.

The effects of quercetin on the different steps of the SGIV life cycle in GS cells were further investigated. The results indicated that quercetin could interfere with SGIV binding to targets on host cells (by 76.14%), disturb SGIV invading into host cells (by 56.03%), and effect SGIV replication in host cells (by 52.73%), respectively. Quercetin had the best antiviral effects during the SGIV life cycle of binding to the receptors on the membrane of host cells, representing the critical stage of SGIV infection. It was consistent with a previous study reported by Ganesan et al. (2012). Rhinovirus (RV) could cause exacerbations in patients with asthma and chronic obstructive pulmonary disease. Ganesan et al. (2012) proved that quercetin could inhibit RV endocytosis and replication. Furthermore, as reported by Wang et al. (2016), (−) epigallocatechin gallate could prevent GCRV from adsorbing and invading host cells by blocking laminin receptor on the surface of host cells, which resulted in its inhibitory effects on grass carp reovirus. We concluded that quercetin could competitively bind to some host cells’ receptors and further prevented SGIV from adsorbing to the host cells. These results were consistent with quercetin’s effects on other viruses (Rojas et al., 2016). Rojas et al. (2016) assessed the effects of quercetin on the different steps of the HCV life cycle and proved that quercetin inhibited HCV mainly through three ways: (i) affecting the virion integrity (by 65%), (ii) decreasing the production of infectious HCV particles, and (iii) reducing the infectivity of newly produced HCV particles (Rojas et al., 2016). Bisignano et al. (2017) determine quercetin’s effects on the replication of HSV-1. It indicated that quercetin not only blocked the production of infectious HSV-1 particles but also inhibited HSV-1 adsorption to cells (Bisignano et al., 2017).

Quercetin belongs to flavonoids. Hoensch and Weigmann (2018) reported that flavonoids could interact with toll-like receptors expressed on the surface of immune cells, then internalize into the cytoplasm and transfer to the nucleus. Then, quercetin could increase the glutathione levels and prevent cell death against an oxidative insult. Furthermore, heat-shock protein 70 (Hsp70) plays a key role in multiple cell functions, including protein translation, folding, intracellular trafficking, and degradation. Hsp70 has been isolated and identified in both nucleocapsids from rabies virus-infected cells and purified virions. Furthermore, Hsp70 also interacts with the nucleoprotein N in host cells (Lahaye et al., 2012). It is speculated that Hsp70 is involved in the different stages of the virus life cycle, including viral transcription, translation, and production. Lahaye et al. (2012) applied quercetin as a specific chaperone inhibitor to down-regulate Hsp70 in rabies virus-infected cells, which resulted in a significant decrease in the number of viral mRNAs, virus proteins, and virus particles (Lahaye et al., 2012). It is interesting to explore the effects of quercetin on Hsp70 regulation during SGIV infection in host cells in a future study.

In summary, *I. verum* extract quercetin appears to have direct and host-mediated antiviral effects against SGIV, which holds great potentials for the development of effective drugs for controlling SGIV infection in aquaculture.

## Data Availability Statement

The raw data supporting the conclusions of this article will be made available by the authors, without undue reservation.

## Author Contributions

PL and QZ conceived and designed the experiments. MLu and QY performed the main experiments. HX and MLi cultured the cells and performed the flow cytometry analysis. YH contributed to the reagents, materials, and analysis tools. All authors contributed to the article and approved the submitted version.

### Conflict of Interest

The authors declare that the research was conducted in the absence of any commercial or financial relationships that could be construed as a potential conflict of interest.
